# A phase I trial of PR-104, a pre-prodrug of the bioreductive prodrug PR-104A, given weekly to solid tumour patients

**DOI:** 10.1186/1471-2407-11-432

**Published:** 2011-10-07

**Authors:** Mark J McKeage, Yongchuan Gu, William R Wilson, Andrew Hill, Karen Amies, Teresa J Melink, Michael B Jameson

**Affiliations:** 1 Auckland Cancer Society Research Centre and Dept of Pharmacology and Clinical Pharmacology, School of Medical Sciences, Faculty of Medical and Health Sciences, The University of Auckland, 89 Grafton Rd, Auckland, New Zealand; 2 Proacta Inc., 4275 Executive Square, Suite 440, La Jolla, CA 92037, USA; 3 Waikato Hospital, Pembroke Street, Hamilton, New Zealand

## Abstract

**Background:**

The phosphate ester PR-104 is rapidly converted in vivo to the alcohol PR-104A, a nitrogen mustard prodrug that is metabolised to hydroxylamine (PR-104H) and amine (PR-104M) DNA crosslinking agents by one-electron reductases in hypoxic cells and by aldo-keto reductase 1C3 independently of oxygen. In a previous phase I study using a q 3 week schedule of PR-104, the maximum tolerated dose (MTD) was 1100 mg/m^2 ^and fatigue, neutropenic fever and infection were dose-limiting. The primary objective of the current study was to determine the dose-limiting toxicity (DLT) and MTD of weekly PR-104.

**Methods:**

Patients with advanced solid tumours received PR-104 as a 1-hour intravenous infusion on days 1, 8 and 15 every 28 days with assessment of pharmacokinetics on cycle 1 day 1. Twenty-six patients (pts) were enrolled (16 male/10 female; median age 58 yrs, range 30 to 70 yrs) who had received a median of two prior chemotherapy regimens (range, 0 to 3) for melanoma (8 pts), colorectal or anal cancer (3 pts), NSCLC (3 pts), sarcoma (3 pts), glioblastoma (2 pts), salivary gland tumours (2 pts) or other solid tumours (5 pts). PR-104 was administered at 135 mg/m^2 ^(3 pts), 270 mg/m^2 ^(6 pts), 540 mg/m^2 ^(6 pts), 675 mg/m^2 ^(7 pts) and 900 mg/m^2 ^(4 pts) for a median of two treatment cycles (range, 1 to 7 cycles) and five infusions (range, 1 to 18) per patient.

**Results:**

Dose-limiting toxicities (DLTs) during cycle one included grade four thrombocytopenia at 540 mg/m^2 ^(1 of 6 pts) and grade four thrombocytopenia and neutropenia at 900 mg/m^2 ^(2 of 4 pts). At an intermediate dose of 675 mg/m^2^, there were no DLTs among a total of seven patients given 12 treatment cycles but all experienced moderate to severe (grade 2 to 4) haematological toxicity. Thrombocytopenia was delayed in its onset and nadir, and its recovery was protracted and incomplete in many patients. There were no complete or partial tumour responses. PR-104-induced thrombocytopenia and neutropenia correlated with plasma AUC of PR-104, PR-104A and an oxidative semi-mustard metabolite (PR-104S1), but no more strongly than with PR-104 dose-level. There was no significant correlation between plasma AUC for the reduced metabolites and myelotoxicity.

**Conclusions:**

Thrombocytopenia, and to a lesser extent neutropenia, was the DLT of weekly PR-104. The MTD was 675 mg/m^2^/week. PR-104 given weekly may be a suitable protocol for further clinical evaluation as a short course of treatment with fractionated radiotherapy or haematopoietic stem cell support, as its duration of dosing is restricted by delayed-onset and protracted thrombocytopenia.

## Background

PR-104 is a phosphate ester dinitrobenzamide mustard pre-prodrug that is rapidly hydrolyzed systemically to PR-104A, a bioreductive prodrug. PR-104A is in turn activated via reduction by NADPH:cytochrome P450 oxidoreductase and other one-electron reductases in hypoxic cells, and by aldo-keto reductase 1C3 (AKR1C3) independently of oxygen, to the corresponding hydroxylamine (PR-104H) and amine (PR-104M) metabolites. Subsequently, these reactive nitrogen mustards crosslink DNA and cause cytotoxicity in cells [1-5]. Hypoxia is a common feature of many solid tumours and is associated with poor prognosis and resistance to conventional radiotherapy and chemotherapy [5-8]. In addition, AKR1C3 has been shown to be up-regulated in several types of human cancer [3,9-11]. Targeting ACK1C3 and hypoxia in tumours is therefore a novel and promising approach to cancer therapy.

PR-104 is known to have preclinical antitumour activity in human tumour xenograft models as mono-therapy and in combination with radiotherapy and chemotherapy [1,2,12]. In rats and dogs its dose-limiting toxicity (DLT) was myelosuppression [13]. A previous phase I study of PR-104 given as a 1-hour intravenous infusion once every three weeks identified fatigue, neutropenic sepsis and infection with normal neutrophil counts as the DLTs and a maximum tolerated dose (MTD) of 1100 mg/m^2 ^3-weekly [13,14]. The current phase I study of weekly PR-104 was undertaken to explore whether its therapeutic index is related to the schedule of administration. If so, then weekly administration may provide higher dose-intensity and greater tumour activation of PR-104, as well as being a schedule potentially suitable for combination with fractionated radiotherapy or with weekly chemotherapy. For this reason, we carried out the phase I study we now report whose primary objective was to determine the MTD of intravenous PR-104 administered weekly in subjects with solid tumours. Secondary objectives included characterising the pharmacokinetics of PR-104, PR-104A and its major metabolites [15,16] and their relationship to toxicity, and assessing evidence of anti-tumour activity.

## Methods

### Patient selection

Eligibility criteria for entry into this study included: age 18 years or more; histologically confirmed malignancy for which no effective therapy existed; measurable or evaluable disease; ECOG Performance Status of 0 or 1; ability to provide written informed consent; no or stable dose of systemic steroid for at least two weeks; adequate bone marrow function (absolute neutrophil count ≥ 1.5 × 10^9^/L, platelet count ≥ 100 × 10^9^/L, haemoglobin level > 90 g/L not maintained by red blood cell transfusion, prothrombin and activated partial thromboplastin times < 1.1 × upper limit of normal (ULN)); adequate liver function (serum bilirubin within normal limits, ALT and AST < 2.5 × ULN), and serum creatinine < 1.5 × ULN. Exclusion criteria included: licensed or investigational anti-cancer therapy (including radiotherapy but excluding androgen deprivation therapy) within four weeks; nitrosoureas or mitomycin C within 6 weeks; prior radiotherapy to more than 25% of bone marrow; prior high-dose chemotherapy; prior receipt of more than three chemotherapy regimens; pregnancy, breast feeding or plans for becoming pregnant during the study; unwillingness to use effective contraception during the study and for 30 days following the last dose of study medication; other medical disorder or laboratory finding that in the opinion of the investigator compromised subject safety; less than four week since major surgery, or; HIV, Hepatitis B surface Antigen or Hepatitis C positivity with abnormal liver function tests.

### Study design

This was a phase I, two-centre, open label, uncontrolled, serial cohort, dose-escalation study evaluating weekly intravenous administration of PR-104. Patients were enrolled in serial cohorts of three patients each using a conventional phase I study design to establish the MTD. Recruitment to dose-levels was expanded if DLT was observed.

### Drug administration

A lyophilized cake of 400 mg of PR-104 was reconstituted in two mL of water for injection, further diluted in 250 mL of 5% dextrose in water and administered as an intravenous infusion over one hour on days 1, 8 and 15 of a 28 day treatment cycle. Prophylactic anti-emetics were administered to all study patients. Other anticancer treatments (except androgen deprivation therapy), prophylactic haematopoietic growth factors and radiotherapy were not permitted during the study.

### Definition of DLT and MTD

Toxicity was assessed according to the National Cancer Institute Common Toxicity Criteria for Adverse Events (version 3.0). DLT was assessed during the first four weeks following the start of PR-104 administration and was defined as any one of the following: grade four thrombocytopenia (platelets < 25 × 10^9^/L) of any duration; other grade four haematological toxicity that lasted for five days or more (haemoglobin < 65 g/L, neutrophils < 0.5 × 10^9^/L); non-haematological toxicity ≥ grade three despite appropriate treatment; neutropenic fever; grade two or higher neurotoxicity lasting one week or more; any toxicity of grade two or higher that has not resolved within two weeks of the end of cycle one (except grade two alopecia). The MTD was defined as a dose level at which one or fewer subjects in six exhibited DLT and for which the next highest dose level demonstrated two or more of six subjects with DLT.

### Starting dose and dose escalation scheme

The starting dose was 135 mg/m^2 ^based on results of toxicology studies in rats and dogs, of which the rat was the most sensitive pre-clinical species to PR-104 (MTD 1328 and 2678 mg/m^2 ^in rats and dogs, respectively by weekly dosing for 4 consecutive weeks). Five dose-levels were explored: 135, 270, 540, 675 and 900 mg/m^2^. Dose-levels were escalated by 100% or less according to the toxicity at preceding dose-levels after review of safety data by the study safety committee.

### Patient evaluation and follow-up

After gaining informed consent, baseline evaluations included a history and physical examination, assessment of performance status, complete blood count (CBC), blood chemistry profile, coagulation studies (INR and APPT), urinalysis, pregnancy test and serum tumour markers. Vital signs and electrocardiogram were taken before, during and after the administration of the first dose of PR-104. With each cycle, weekly assessments included performance status, symptom-directed physical examination, laboratory investigations (CBC, coagulation studies, serum chemistry and urinalysis), intercurrent adverse events and concomitant medication use. Extent of disease was determined by computed tomography or magnetic resonance scans prior to enrolment and repeated once every two treatment cycles. Efficacy was assessed using Response Evaluation Criteria in Solid Tumours (RECIST) criteria version 1.0 [17]. Where possible, complete blood counts data was collected after the end of the study participation until death or commencement of other cancer therapy.

### Pharmacokinetic analyses

To evaluate the pharmacokinetics of PR-104, PR-104A and their major metabolites, blood samples were collected in EDTA vacutainer tubes pre-dose, 45 minutes into the infusion, immediately following the completion of the infusion and at 5, 10, 20, 30, 45, 60, 120, 240 minutes and 24 (±8) hours post-infusion of the first dose. Blood samples were centrifuged for five minutes to prepare plasma. Plasma was then immediately deproteinised by addition of nine volumes of methanol:ammonium acetate: acetic acid (1000:3.5:0.2 v/w/v) and stored at -70°C. Extracts were assayed by validated ultra high-performance liquid chromatography methods [18] using triple quadrupole mass spectrometric detection with tetradeuterated internal standards [19]. Non-compartmental pharmacokinetic analyses using WinNonLin (v4.0.1), and actual infusion times and doses, was used to derive the maximum plasma concentration (C_max_), area under the plasma concentration time curve extrapolated to infinity (AUC_(0-inf)_), clearance (Cl), volume of distribution at steady state (V_ss_) and elimination half-life (t_1/2_).

### Statistics

Data were analysed using descriptive statistics including the median, range and proportion, and mean and standard deviation for normally distributed data. Relationships between dose-levels, pharmacokinetic parameters, percentage change in platelet and neutrophil counts and creatinine clearance were assessed by nonparametric correlation analysis. A *P *< 0.05 was regarded as being statistically significant.

## Results

### Patient characteristics and study treatment

This study enrolled a total of 26 adult cancer patients (16 males, 10 females), ranging in age from 30 to 70 years (median, 58 years) with cancer diagnoses including melanoma (8 patients), colon, rectum or anus cancer (3 patients), non-small cell lung cancer (NSCLC) (3 patients), sarcoma (3 patients), glioblastoma (2 patients), salivary gland tumours (2 patients) or other tumour types (5 patients) (Table 1). All but four patients (15%) had received prior chemotherapy. Cohorts of three to seven patients were treated at one of five dose-levels (135, 270, 540, 675 and 900 mg/m^2^) of PR-104 given by intravenous infusion on days 1, 8 and 15 repeated every 28 days (Table 2). Patients received a median of two treatment cycles (range, 1 to 7) and five infusions (range, 1 to 18) of PR-104.

**Table 1:** Patient characteristics and previous chemotherapy

Characteristic	Number of patients (%)
**Gender**	
**Male**	16 (62)
**Female**	10 (38)
**Age (years)**	
**Mean (range)**	58 (30-70)
**ECOG Performance Status**	
**0**	8 (31)
**1**	18 (69)
**Tumour Type**	
**Melanoma**	8 (31)
**Colon, rectum or anus**	3 (12)
**Non-small cell lung**	3 (12)
**Sarcoma**	3 (12)
**Glioblastoma**	2 (8)
**Salivary gland**	2 (8)
**Adrenal Cortex**	1 (4)
**Cervix**	1 (4)
**Head and neck**	1 (4)
**Kidney**	1 (4)
**Pancreas**	1 (4)
**Previous chemotherapy**	
**No. of courses**	
**None**	4 (15)
**One**	8 (31)
**Two**	7 (27)
**Three**	7 (27)

**Table 2:** Dose escalation scheme, treatment delivery and cycle-one dose-limiting toxicity (DLTs)

Cohort Number	Dose level (mg/m2/wk)	Dose increment	Number of patients	Total number of cycles (median per patient)	Total number of infusions (median per patient)	Number of patients with cycle-one DLT
**1**	135	-	3	9 (2)	32 (6)	0
**2**	270	100%	6	17 (2)	50 (6)	0
**3**	540	100%	6	11 (2)	30 (5)	1^a^
**4**	675	25%	7	12 (2)	33 (5)	0
**5**	900	33%	4	6 (1.5)	15 (4)	2^b^

^a^grade 4 thrombocytopenia; ^b^grade 4 thrombocytopenia and grade 4 neutropenia lasting > 5 day

### MTD determination and DLTs

The MTD of weekly PR-104 was 675 mg/m^2 ^(Table 2). Cycle one DLTs were experienced by two of four patients (50%) receiving 900 mg/m^2^, which was one dose-level higher than the MTD, and by one of six patients (17%) receiving 540 mg/m^2^, which was one dose-level lower than the MTD. One DLT was experienced by a 44 year old male, with glioblastoma previously treated with two lines of chemotherapy, who developed grade 4 neutropenia on day 14 of cycle one of PR-104 900 mg/m^2 ^lasting greater than five days. A second DLT occurred in a 66 year old female, with NSCLC previously treated with two lines of cytotoxic chemotherapy and with erlotinib, who developed grade 4 thrombocytopenia after going off-study for progressive disease after a single infusion of PR-104 at 900 mg/m^2^. A third DLT occurred in a 55 year old male, with metastatic melanoma previously treated with radiotherapy and dacarbazine, who developed new cerebral metastases and extra-cranial progressive disease leading him to go off-study after the first treatment cycle of PR-104 at 540 mg/m^2^. Twenty days later, he presented with headache, vomiting, seizures and a platelet count of 22 × 10^9^/L. A CT scan showed intra-cerebral, subarachnoid and intracranial tumour haemorrhage. The patient died five days later despite supportive measures. At the intermediate dose of 675 mg/m^2^, there were no cycle one DLTs in a total of seven patients.

### Haematological toxicity

**Figure 1:**
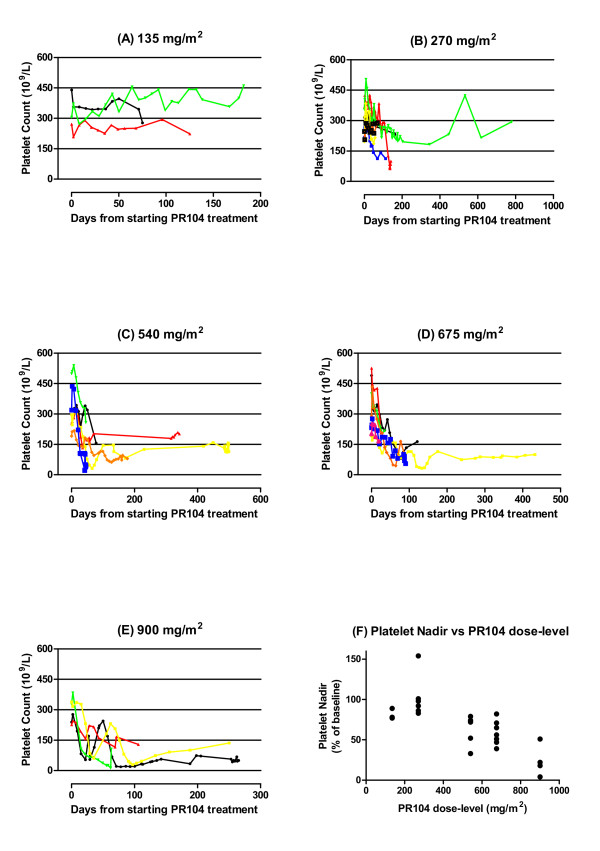
**PR-104-induced thrombocytopenia followed until death or further treatment**. (a) Time-course of thrombocytopenia in each subject at 135 mg/m^2 ^(b) Time-course of thrombocytopenia in each subject at 240 mg/m^2 ^(c) Time-course of thrombocytopenia in each subject at 540 mg/m^2 ^(d) Time-course of thrombocytopenia in each subject at 675 mg/m^2 ^(e) Time-course of thrombocytopenia in each subject at 900 mg/m^2 ^(f) Platelet nadir on first treatment cycle correlated with PR-104 dose-level (Spearman r = -0.79; *P *< 2 × 10^-7^).

**Figure 2:**
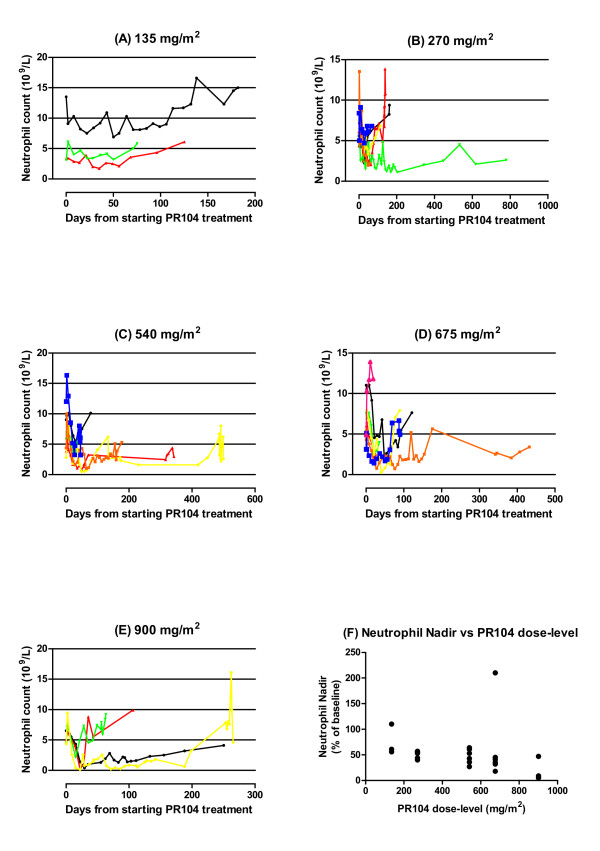
**PR-104-induced neutropenia followed until death or further treatment**. (a) Time-course of neutropenia in each subject at 135 mg/m^2 ^(b) ime-course of neutropenia in each subject at 240 mg/m^2 ^(c) Time-course of neutropenia in each subject at 540 mg/m^2 ^(d) Time-course of neutropenia in each subject at 675 mg/m^2 ^(e) Time-course of neutropenia in each subject at 900 mg/m^2 ^(f) Neutrophil nadir on first treatment cycle correlated with PR-104 dose-level (Spearman r = -0.56; *P *< 0.005)

Haematological toxicity was the most significant type of toxicity in this study. Of 26 patients treated with weekly PR-104, 23 or 88% experienced some form of moderate to severe haematological toxicity (grade ≥ 2) (Table 3), and haematological toxicity DLTs defined the MTD, as outlined above. Further occurrences of haematological toxicity are outlined below, in Table 3, and in Figures 1 and 2.

**Table 3:** Haematological toxicity

Worst toxicity severity grade of haematological toxicity by patient (number of patients (%))																		
Dose level	135 mg/m^2^			270 mg/m^2^			540 mg/m^2^			675 mg/m^2^			900 mg/m^2^			All doses		
Number of patients	n = 3			n = 6			n = 6			n = 7			n = 4			n = 26		
						
Toxicity severity grade	2	3	4	2	3	4	2	3	4	2	3	4	2	3	4	2	3	4
Anaemia	1 (33)	1 (33)		2 (33)	1 (17)		4 (67)	1 (17)		4 (57)	1 (14)	1 (14)	2 (50)	2 (50)		13 (50)	6 (23)	1 (14)
Leukopenia				1 (17)			2 (33)	1 (17)		3 (43)	2 (29)		2 (50)	1 (25)		8 (31)	4 (15)	
Neutropenia				1 (17)			2 (33)	1 (17)		2 (29)	1 (14)				3 (75)	5 (19)	2 (8)	3 (12)
Thrombocytopenia				1 (17)			1 (17)	1 (17)	1 (17)	1 (14)	2 (29)			1 (25)	2 (50)	3 (12)	4 (15)	2 (8)
Any haematological toxicity	1 (33)	1 (33)		3 (50)	1 (17)		3 (50)	2 (33)	1 (17)	4 (57)	2 (29)	1 (14)			4 (100)	11 (42)	6 (23)	6 (23)

Thrombocytopenia occurred in 14 of 26 patients (54%) treated with weekly PR-104 and ranged from grade 1 to 4 in severity (Table 3). The incidence of moderately-severe or severe thrombocytopenia (grade 3 or 4) was 0, 33, 29 and 75% at the ≤ 270, 540, 675 and 900 mg/m^2 ^dose-levels, respectively. Onset of thrombocytopenia was delayed by a median of six weeks (range 2 to 18 weeks), and thrombocytopenia nadirs by a median of ten weeks (range 5 to 19 weeks), relative to the start of PR-104 treatment (Figure 1A-E). The maximum percentage change in platelet count from baseline following the first treatment cycle correlated strongly with PR-104 dose-level (Spearman r = -0.79; *P *= 2 × 10^-7^) (Figure 1F). Platelet counts did not recover to baseline levels in any patients who had experienced thrombocytopenia of any severity grade. Sustained recovery of platelet counts to within normal limits occurred in only two of 14 patients (14%) who had experienced thrombocytopenia from PR-104. The duration of thrombocytopenia was correspondingly protracted and incompletely defined (median, at least 7 weeks; range, 1 to at least 64 weeks), due to the persistence of this toxicity until the death of study patients or their commencement of further treatment, which confounded interpretation of subsequent blood counts. Platelet transfusions were required for severe thrombocytopenia and/or bleeding by some patients but specific details were lacking because data collection on blood product support data was not part of the prospective study plan.

Neutropenia occurred in 11 of 26 patients (42%) and ranged from grade 1 to 4 in severity (Table 3). Seven patients had more than one episode of neutropenia associated with repeated cycles of treatment. The incidence of moderately-severe to severe neutropenia (grade 3 to 4) was 11, 17, 14 and 75% at the ≤ 270, 540, 675 and 900 mg/m^2 ^dose-levels, respectively. Onset of the first episode of neutropenia occurred after a median of three weeks (range 2 to 5 weeks), and nadirs of first episode of neutropenia occurred after a median of four weeks (range 3 to 7 weeks), relative to the time of starting PR-104 treatment (Figure 2A-E). Maximum percentage change in neutrophil count from baseline following the first treatment cycle correlated with PR-104 dose-level (Spearman r = -0.56; *P *= 0.005) (Figure 2F). The duration of the first episode of neutropenia ranged from one to 42 days (median 7 days). Subsequent episodes of neutropenia tended to be more severe and longer lasting than the initial occurrence especially when no dose reductions or omissions were made for prior neutropenia. Neutrophil counts recovered to baseline levels and/or to within normal limits in all patients who had experienced neutropenia from PR-104. There was one uncomplicated episode of neutropenic sepsis.

Anaemia occurred in 24 of 26 patients (92%), ranged from grade 1 to 4 in severity and was managed by red cell transfusion. The incidence of moderately-severe or severe anaemia (grade 3 or 4) was 30, 17, 17, 28 and 50% at the 135, 270, 540, 675 and 900 mg/m^2 ^dose-levels, respectively. There was no correlation between maximum percentage change in haemoglobin level from baseline and PR-104 dose-level.

### Non-haematological toxicity

Fatigue was the most common non-haematological toxicity that was considered related to PR-104 treatment (Table 4). Gastrointestinal symptoms were reported by about one quarter of patients but were self-limiting and often considered unrelated to PR-104 treatment. Neither were strongly related to dose level.

**Table 4:** Non-haematological toxicity

Worst toxicity severity grade of non-haematological toxicity by patient (number of patients (%))			
**Toxicity severity grade**	2	3	4
**Fatigue**	10 (38)	2 (8)	
**Abdominal pain**	4 (15)	2 (8)	
**Constipation**	7 (27)		
**Nausea**	5 (19)	2 (8)	
**Back pain**	3 (12)	2 (8)	
**Oedema peripheral**	5 (19)		
**Vomiting**	3 (12)	2 (8)	

### Tumour response

**Figure 3:**
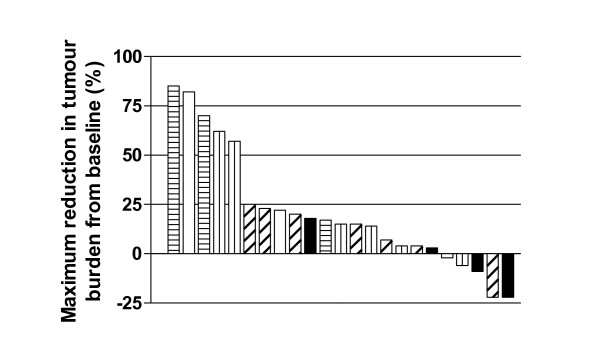
**Waterfall plot of maximum reduction in tumour burden from baseline by dose level (135 mg/m**^**2 **^**(white fill), 270 mg/m**^**2 **^**(vertical lines), 540 mg/m**^**2 **^**(diagonal lines), 675 mg/m**^**2 **^**(horizonal lines) and 900 mg/m**^**2 **^**(black fill))**.

There were no complete or partial responses. Best RECIST response category was progressive disease in 16 patients (61%), stable disease in eight patients (31%) and non-evaluable in two patients (8%). A waterfall plot showed that tumour dimensions increased during PR-104 treatment in most patients (Figure 3). Two patients, one each with soft tissue sarcoma (675 mg/m^2^)and glioblastoma (900 mg/m^2^), showed 22% reduction in tumour burden from baseline but neither fulfilled RECIST criteria for partial response.

### Pharmacokinetics

**Figure 4:**
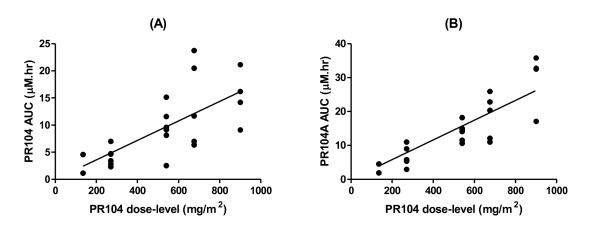
**Linear relationship between PR-104 dose-level and PR-104 (A) and PR-104A exposure (B)**.

Cmax and AUC values for PR-104 and PR-104A increased in approximate proportion to respective dosage increments between different dose-levels (Table 5), and were linearly related to PR-104 dose-level (Figure 4). PR-104 clearance was high (mean Cl, 107 L/h/m^2^; CV, 70%). PR-104 volume of distribution approximated total body water (mean Vd_ss_, 35.8 L/m^2^; CV, 63%). PR-104 half life was short (mean t_1/2_, 0.10 h; CV, 75%), while PR-104A had a longer half life (mean t_1/2_, 0.49 h; CV, 31%). PK parameters for the major plasma metabolites derived from PR-104A (glucuronide PR-104G, reduced metabolites PR-104H and PR-104M, and oxidative metabolite PR-104S1) are shown in Table 6. Exposure (AUC) was greatest for the glucuronide PR-104G, followed by hydroxylamine PR-104H, with lower AUC values for PR-104M and PR-104S1. These metabolites had plasma half lives comparable to PR-104A (mean t_1/2 _values 0.43, 0.61, 0.70 and 0.79 h respectively), suggesting that their clearance may be rapid relative to the kinetics of their formation. Plasma AUCs for PR-104, PR-104A and metabolites appeared similar on cycle 1 day 1 to cycle 1 day 8 in three patients who had pharmacokinetic studies on repeated dosing at the MTD (675 mg/m^2^) (Table 7). One patient given naproxen, an inhibitor of AKR1C3 [20] and a substrate for UGT2B7 [21], on cycle 2 day 1 (500 mg orally two hours prior to PR-104) showed no apparent associated change in the reduction or glucuronidation of PR-104A compared to cycle 1 day 1.

**Table 5:** PR-104 and PR-104A plasma pharmacokinetic parameters for cycle 1 day 1

			**C**_**max **_**(μM)**		AUC (μM.h)		**t**_**1/2 **_**(h)**		**Cl (L/h/m**^**2**^**)**	**Vd**_**ss **_**(L/m**^**2**^**)**
**Dose-level (mg/m**^**2**^**)**		n	PR-104	PR-104A	PR-104	PR-104A	PR-104	PR-104A	PR-104	PR-104
										
**135**		2	4.08	3.49	2.85	3.22	0.06	0.34	128	38.7
**270**		6	7.63 (3.13)	6.81 (2.15)	4.15 (1.69)	6.61 (2.88)	0.07 (0.05)	0.42 (0.16)	129 (49.4)	39.1 (9.83)
**540**		6	14.2 (6.51)	13.7 (2.94)	9.34 (4.17)	14.0 (2.70)	0.07 (0.03)	0.42 (0.16)	130 (119)	44.7 (38.5)
**675**		7	18.1 (9.9)	15.5 (6.1)	12.9 (7.0)	17.7 (5.6)	0.14 (0.10)	0.55 (0.11)	92.7 (52.2)	27.3 (14.4)
**900**		4	20.8 (5.71)	23.3 (4.64)	15.2 (4.97)	29.5 (8.44)	0.15 (0.10)	0.68 (0.14)	64.8 (22.9)	24.2 (10.5)

Values are the mean (SD)

**Table 6:** Plasma pharmacokinetic parameters for PR-104A metabolites, cycle 1 day 1

		**C**_**max **_**(μM)**				AUC (μM h)				**t**_**1/2 **_**(h)**			
**Dose-level (mg/m**^**2**^**)**	n	PR-104G	PR-104H	PR-104M	PR-104S1	PR-104G	PR-104H	PR-104M	PR-104S1	PR-104G	PR-104H	PR-104M	PR-104S1
**135**	2	1.66	0.66	0.05	0.06	1.67	0.79	0.08	0.10	0.25	0.48	0.53	0.76
**270**	6	3.02 (2.12)	0.68 (0.29)	0.06 (0.02)	0.14 (0.09)	3.96 (3.81)	0.81 (0.37)	0.11 (0.05)	0.25 (0.17)	0.40 (0.22)	0.51 (0.12)	0.88 (0.43)	1.09 (0.51)
**540**	6	11.27 (8.07)	0.49 (0.17)	0.07 (0.02)	0.20 (0.08)	13.85 (12.01)	0.63 (0.26)	0.11 (0.04)	0.29 (0.13)	0.38 (0.14)	0.41 (0.12)	0.56 (0.15)	0.59 (0.23)
**675**	7	8.71 (4.52)	0.51 (0.27)	0.08 (0.05)	0.36 (0.25)	11.9 (7.4)	0.71 (0.32)	0.13 (0.06)	0.49 (0.45)	0.49 (0.17)	0.74 (0.37)	0.75 (0.28)	0.68 (0.12)
**900**	4	7.81 (3.92)	0.39 (0.16)	0.07 (0.03)	0.31 (0.13)	11.19 (6.54)	0.69 (0.26)	0.16 (0.08)	0.64 (0.25)	0.68 (0.18)	0.85 (0.09)	0.88 (0.11)	0.79 (0.15)

Values are the mean (SD)

**Table 7:** Lack of change in plasma AUCs (μM.h) of PR-104, PR-104A and metabolites with repeat dosing of PR-104 at 675 mg/m^2 ^on cycle 1 day 1 (C1D1), cycle 1 day 8 (C1D8) and cycle 2 day 1 (C2D1)

Patient	Cycle	PR-104	PR-104A	PR-104G	PR-104H	PR-104M	PR-104S1
**04-75**	C1D1	6.34	12.11	6.60	0.51	0.06	0.16
	C1D8	6.61	13.16	7.74	0.44	0.09	0.16
**01-76**	C1D1	6.76	14.14	21.38	0.56	0.11	0.87
	C1D8	7.91	16.72	28.01	0.45	0.10	1.09
**04-77**	C1D1	14.55	17.81	7.07	0.25	0.12	0.31
	C1D8	11.01	16.96	7.53	0.26	0.12	0.36
	C2D1*	8.16	19.53	6.73	0.59	0.10	0.44

*, given with naproxen.

Exposure to PR-104, PR-104A and PR-104G (AUC values) correlated significantly with the severity of thrombocytopenia and neutropenia (Table 8), although the correlation coefficients were no higher than for the relationships between toxicity and PR-104 dose. Notably, the AUC for the cytotoxic reduced metabolites PR-104H and PR-104M (combined in Table 8) did not correlate significantly with either thrombocytopenia or neutropenia.

**Table 8:** Correlations between drug exposure and toxicity, normalised drug exposure and creatinine clearance (Spearman rank order analysis).

Exposure	**platelets**^**†**^	**neutrophils**^**†**^	**CrCl**^**‡**^
**Dose**	-0.79 (2 × 10^-7^)	-0.56 (5 × 10^-3^)	-
**PR-104 AUC**	-0.56 (0.005)	-0.35 (0.1)	-0.14 (0.52)
**PR-104A AUC**	-0.75 (2 × 10^-7^)	-0.49 (0.02)	-0.12 (0.59)
**PR-104G AUC**	-0.51 (0.01)	-0.13 (0.56)	-0.01 (0.98)
**PR-104(H+M) AUC**	0.09 (0.67)	-0.04 (0.84)	-0.04 (0.86)
**PR-104S1 AUC**	-0.61 (0.002)	-0.53 (0.009)	-0.16 (0.46)

^†^At nadir (percentage of pretreatment value)

^‡^Creatinine clearance. Drug exposures (AUC) are normalised by dose levels.

Values are correlation coefficient and (p value).

## Discussion

We describe a phase I trial evaluating weekly administration of PR-104, a novel nitrogen mustard pro-drug activated by hypoxia and AKR1C3. Tumour hypoxia is common in solid tumours and associated with poor prognosis and resistance to radiotherapy and chemotherapy [6-8]. AKR1C3 has been shown to be up-regulated in some human cancers [9-11]. On this basis, targeting tumour hypoxia and AKR1C3 is a promising approach to cancer therapy.

In the current study, we determined the MTD of weekly PR-104 to be 675 mg/m^2 ^given on days 1, 8 and 15 every 28 days. This MTD was established by the occurrence of protocol-defined DLTs in one of six (17%) of patients at the 540 mg/m^2 ^dose-level, and in two of four (50%) of patients at the 900 mg/m^2 ^dose-level. DLTs included two episodes of grade four thrombocytopenia, and a single episode of grade four neutropenia lasting greater than five days. In contrast, there was no DLT at the MTD dose-level in a total of seven patients given a total of 12 treatment cycles (median 2, per patient) and 33 infusions (median, 5 per patient), although all seven experienced moderate to severe (grade 2 to 4) haematological toxicity. Thus, this study met its primary objective of determining the DLT and MTD of weekly PR-104.

Thrombocytopenia was the most significant clinical toxicity associated with weekly PR-104 in this phase I study. It was the major DLT and necessitated platelet transfusions for very low counts and/or bleeding in some patients but specific details of blood product support were not collected prospectively. The onset and nadir of thrombocytopenia were delayed relative to the commencement of PR-104 treatment by a median of six and ten weeks, respectively. Thereafter, thrombocytopenia persisted without recovery to baseline or to normal levels in most patients in whom it was feasible to undertake longer term monitoring of platelet counts. Persisting thrombocytopenia limited the delivery of weekly treatment with PR-104 beyond two treatment cycles at dose-levels of 540 mg/m^2 ^or more. The extent and significance of protracted thrombocytopenia may have been underestimated by this and previous studies of PR-104 because of their use of definitions for DLTs confined to adverse-events occurring within the first four weeks of treatment, their lack of prospectively defined plans for longer term monitoring of haematological toxicity and difficulties doing so in study populations with short survival. In contrast, neutropenia was earlier in onset and more reversible than thrombocytopenia but also common and dose-limiting. Other toxicities included anaemia, fatigue and gastrointestinal symptoms but were less clinically significant and not dose-limiting.

One hypothesis for the mechanism underlying the myelotoxicity of PR-104 is that the circulating reduced metabolites (PR-104H and PR-104M), which are activated nitrogen mustards, might be responsible. However, the lack of correlation between systemic exposure to these metabolites and either neutropenia or thrombocytopenia (Table 8) argues against this hypothesis. This in turn suggests that myelotoxicity might result from activation of PR-104A locally in the bone marrow, as a result of expression of AKR1C3 in myeloid progenitor cells [22] and/or hypoxia in bone marrow stem cell niches as reported in mice [23].

The findings of this study suggest that treatment protocols for giving PR-104 at weekly intervals at or about a dose of 675 mg/m^2 ^may be feasible for a short course of treatment only as the total duration of treatment would be restricted by toxicity. Continued treatment at this dose-level beyond six to eight weeks may not be feasible because of delayed onset thrombocytopenia that would then persist, necessitating the deferral, reduction or omission of subsequent PR-104 dosing. Single doses of PR-104 of 675 mg/m^2 ^were shown by pharmacokinetic studies carried out on day one of cycle one in the current study to achieve systemic exposures to PR-104A ranging between 10.9 to 25.9 uM.hr. These PR-104A AUC values were close to but lower than those (> 30 μM.hr) achieved in a previous phase I study of q 3 weekly dosing of PR-104 [14], and those associated with preclinical activity of PR-104 in human tumour xenograft models (> 30 μM.hr) [1,2,12]. Repeated dosing of PR-104 at 675 mg/m^2 ^was shown to achieve cumulative exposures to PR-104A exceeding these threshold levels required for experimental activity, since a target AUC of 30 μM.hr or more was exceeded after two or three doses (Table 7). In addition, reductions in tumour dimensions of > 20% seen in two patients at > 540 mg/m^2^, and significant myelosuppression likely related to the main pharmacological action of PR-104, provides further supporting evidence for therapeutically active exposures having been achieved on the phase I trial of weekly treatment. Thus, treatment protocols for giving PR-104 weekly at or about a dose of 675 mg/m^2 ^over 4 to 6 weeks may be suitable for future study to explore its clinical feasibility and activity as a single agent or in combination therapy.

Short courses of weekly PR-104 may be suitable for use concurrently with fractionated radiotherapy or as high-dose chemotherapy prior to bone marrow reconstitution. Many conventional fractionated radiotherapy regimens are limited by tumour hypoxia and given over a short period of up to six weeks, to which it may be feasible to add weekly PR-104. The delayed-onset myelosuppresion of PR-104, combined with its minimal non-haematological toxicity, may allow its delivery at or beyond the current MTD prior to haematological stem cell support via infusions of autologous bone marrow or peripheral blood stem cells. It will be necessary however to establish the feasibility of treatment protocols based on weekly PR-104 combined with fractionated radiotherapy and/or bone support in further early phase clinical studies with careful longer term monitoring of haematological toxicity and provision of blood product support as required. An exploratory study of PR-104 in relapsed and treatment-refractory acute leukaemia is currently underway (ClinicalTrials.gov Identifier: NCT01037556; http://clinicaltrials.gov/ct2/show/NCT01358227?term=pr104&rank=3) that is supported by evidence of preclinical activity in this disease setting [24].

## Conclusions

This study identified thrombocytopenia, and to a lesser extent neutropenia, as the DLT of weekly PR-104, and its MTD as 675 mg/m^2^/week. Weekly PR-104 may be a suitable administration schedule for further evaluation as a short treatment course in combination with fractionated radiotherapy or prior to haematopoietic stem cell support.

## Competing interests

WRW is a stock holder and advisor to Proacta, Inc. TJM is an employee of Proacta, Inc. The authors have no other competing interests to declare.

## Authors' contributions

MJM contributed to the study design, patient recruitment, clinical study procedures, data interpretation and preparation of the final manuscript. YG and WRW contributed to the pharmacokinetic study procedures, data interpretation and preparation of the final manuscript. AH and KA contributed to patient recruitment and clinical study procedures. TJM contributed to the study design, data interpretation and preparation of the final manuscript. MBJ contributed to the study design, patient recruitment, clinical study procedures, data interpretation and preparation of the final manuscript. All authors read and approved the final manuscript.

## Pre-publication history

The pre-publication history for this paper can be accessed here:

http://www.biomedcentral.com/1471-2407/11/432/prepub
